# Plug-and-Play PRNU Enhancement Algorithm with Guided Filtering

**DOI:** 10.3390/s24237701

**Published:** 2024-12-02

**Authors:** Yufei Liu, Yanhui Xiao, Huawei Tian

**Affiliations:** School of National Security, People’s Public Security University of China, Beijing 100038, China; 2022211246@stu.ppsuc.edu.cn (Y.L.); hwtian@ppsuc.edu.cn (H.T.)

**Keywords:** digital imaging-device forensic, source camera identification, photo response non-uniformity, guided filtering, high-frequency enhancement

## Abstract

As a weak high-frequency signal embedded in digital images, Photo Response Non-Uniformity (PRNU) is particularly vulnerable to interference from low-frequency components during the extraction process, which affects its reliability in real-world forensic applications. Previous studies have not successfully identified the effective frequency band of PRNU, leaving low-frequency interference insufficiently suppressed and impacting PRNU’s utility in scenarios such as source camera identification, image integrity verification, and identity verification. Additionally, due to differing operational mechanisms, current mainstream PRNU enhancement algorithms cannot be integrated to improve their performance further. To address these issues, we conducted a frequency-by-frequency analysis of the estimated PRNU and discovered that it predominantly resides in the frequency band above 10 Hz. Based on this finding, we propose a guided-filtering PRNU enhancement algorithm. This algorithm can function as a plug-and-play module, seamlessly integrating with existing mainstream enhancement techniques to further boost PRNU performance. Specifically, we use the PRNU components below 10 Hz as a guide image and apply guided filtering to reconstruct the low-frequency interference components. By filtering out these low-frequency components, we retain and enhance the high-frequency PRNU signal. By setting appropriate enhancement coefficients, the low-frequency interference is suppressed, and the high-frequency components are further amplified. Extensive experiments on publicly available Dresden and Daxing digital device forensics datasets confirm the efficiency and robustness of the proposed method, making it highly suitable for reliable forensic analysis in practical settings.

## 1. Introduction

With the rapid expansion of mobile internet and the widespread use of imaging devices like smartphones, digital images have become a crucial information medium on social media platforms. Meanwhile, accessible editing software such as Photoshop, Lightroom, Canva, and Meitu has lowered the barrier to image manipulation, enabling malicious actors to alter and distribute images for illicit purposes. Ensuring image authenticity and integrity has thus become a priority in areas like forensic identification and criminal investigation [1]. In the field of digital image forensics, Source Camera Identification (SCI) based on Photo Response Non-Uniformity (PRNU) has received considerable attention. PRNU is a unique, physical “fingerprint” embedded in digital images due to sensor manufacturing defects and silicon non-uniformity. Comparing PRNU allows for the verification of image sources, often referred to as the “fingerprint” of imaging devices due to their uniqueness, ubiquity, and stability [2]. PRNU has even been accepted as evidence in U.S. courts to verify the source and integrity of digital images [3,4,5] and holds promise for identity verification tasks as well [6]. This paper primarily explores PRNU’s application in SCI tasks.

PRNU manifests in digital images as a common high-frequency pattern noise [7], making it possible to estimate by calculating the common components of the image’s noise residuals [2]. According to [8], a more effective noise extraction algorithm can extract noise residuals that contain more PRNU components. Therefore, choosing a noise extraction algorithm is crucial for PRNU extraction. As a result, many studies have focused on developing noise extraction algorithms with better performance to extract the PRNU as comprehensively as possible [9,10,11,12]. Among these studies, Cortiana et al. [9] designed a PRNU extraction method based on the BM3D noise extraction algorithm, achieving promising results. To address the complexity and long computational time of BM3D, Zeng et al. [10] propose a PRNU extraction method based on content-adaptive guided image filtering. This method achieves performance comparable to the BM3D-based method but with significantly reduced complexity. Later, to alleviate the difficulty of noise extraction in regions around strong edges of images, Zeng et al. [11] introduce a method based on dual-tree complex wavelet transform to extract the PRNU from a given image. This method outperforms the BM3D algorithm in regions around strong edges. With the rise of deep learning technologies, noise extraction methods based on deep neural networks have achieved significant success, further advancing PRNU extraction techniques. One representative example is [12], which proposes an effective PRNU fingerprint extraction algorithm based on a densely connected hierarchical denoising network (DHDN). DHDN can more effectively capture real-world noise, so DHDN-based PRNU extraction methods have significantly outperformed BM3D-based methods in SCI tasks.

The aforementioned studies focus on improving noise extraction techniques to preserve the integrity of PRNU as much as possible. However, digital imaging post-processing pipelines inherently introduce various types of low-frequency interference that can obscure or distort the Photo Response Non-Uniformity (PRNU) signal, which is primarily high-frequency. These low-frequency interferences include sensor-induced artifacts, demosaicing and compression effects, et al. [6,13]. Such interferences often overlap with or even mask the PRNU signal, making it difficult to extract an accurate PRNU profile without interference. But, noise extraction algorithms are too coarse [14] and are unable to effectively filter out these low-frequency interference signals [15].

Therefore, many studies not only work on improving noise extraction algorithms but also explore methods to enhance PRNU [4,16,17]. In one of these studies, Chen et al. [4] propose a Removing the Sharing Components (RSC) method, which uses zero-mean and Wiener filtering operations to eliminate both periodic and non-periodic Non-Unique Artifacts (NUAs), resulting in a smoother PRNU. Based on the assumption that PRNU follows a Gaussian distribution, Lin et al. [16] propose the Spectrum Equalization Algorithm (SEA), which enhances the PRNU components by suppressing anomalous peaks in the Fourier transform domain. Due to their ease of use and effectiveness, RSC and SEA have become the most widely used PRNU enhancement algorithms. Furthermore, Rao et al. [17] introduce a Principal Component Analysis (PCA)-based method for suppressing random artifacts, naming it the DC method. This method shows improved performance over SEA. However, these enhancement methods overlook the fact that PRNU is a high-frequency signal and, therefore, retains low-frequency interference. Additionally, due to differences in their mechanisms, these methods cannot be integrated to further enhance PRNU performance. To address this limitation, Gupta et al. [15] propose a method that applies Discrete Cosine Transform (DCT) to PRNU after SEA processing. By setting appropriate thresholds in the DCT domain, the low-frequency components are directly filtered out, leading to further enhancement of PRNU. However, this method is not ideal as a flexible plug-and-play module because it does not clearly define the range of low-frequency components and simply removes all of them, requiring the selection of appropriate hyper-parameters based on specific circumstances. Moreover, directly subtracting low-frequency components to suppress them introduces a large sampling error.

Based on the above analysis, to address the issue of PRNU being affected by low-frequency interference and the limitations of current enhancement algorithms that cannot be integrated due to the unknown characteristics of PRNU itself, we propose a universal and efficient PRNU enhancement scheme, focusing on the intrinsic properties of PRNU. The main contributions of this paper can be summarized in the following two aspects:

- We conduct a comprehensive frequency-by-frequency analysis of PRNU to identify its primary frequency range, offering new insights into the spectral characteristics of PRNU and its vulnerability to low-frequency interference;
- We propose a novel guided-filtering PRNU enhancement algorithm that effectively reconstructs and eliminates low-frequency interference, enhancing the high-frequency PRNU components. This algorithm can be seamlessly integrated with existing mainstream enhancement techniques as a plug-and-play module, ensuring improved PRNU performance with low computational complexity.

The paper is organized as follows: Section 2 introduces the related works about the research object of this paper. Section 3 details the proposed guided-filtering PRNU enhancement algorithm. Section 4 conducts extensive experiments to evaluate the performance of the proposed algorithm. Finally, Section 5 concludes this paper.

## 2. Related Work

As a physically unclonable hardware fingerprint, PRNU is widely employed for SCI tasks at the individual device level. This section summarizes two key research areas: “hardware fingerprint” and “source camera identification”.

### 2.1. Hardware Fingerprint

Hardware fingerprint-based identification utilizes unique physical characteristics inherent in device components, which are universal, unique, permanent, and measurable [18]. Beyond PRNU, researchers have explored other hardware fingerprints, including RF, MEMS, and audio fingerprints. Each of these fingerprints leverages the physical variations generated during manufacturing, making them secure, unclonable, and difficult to tamper with.

For example, RF fingerprints exploit device-specific signal variations during wireless transmission [19,20], while MEMS fingerprints leverage minor discrepancies in sensor outputs (e.g., from gyroscopes and accelerometers) for mobile device verification [21]. Audio fingerprints similarly capture slight physical differences in microphones and speakers as identifiable features in recorded or played audio [22]. However, since these hardware fingerprints are indirectly analyzed through output signals, noise suppression remains critical for achieving reliable identification [23].

### 2.2. Source Camera Identification

Research on SCI can be categorized into device model-level and individual device-level identification. Model-level methods leverage unique hardware and software characteristics of device models, such as lens distortion, color filter array (CFA) patterns, and compression parameters [24,25,26,27,28,29,30]. Among these, CFA features are particularly effective due to their robustness and distinguishability [31]. Additionally, some research focuses on extracting statistical features from images to effectively distinguish device models [32,33].

Individual device-level SCI focuses on identifying specific devices within the same model, often using PRNU as the most reliable feature [31,34,35]. Other approaches, such as dark spots, dead pixels, and dark current noise, have been explored but face challenges in robustness and practicality [36,37,38]. Recently, deep learning-based SCI has shown promise, achieving notable improvements in both model and device-level tasks. However, these methods often rely on closed datasets, limiting their applicability in open environments, where PRNU continues to be a valuable tool [39,40].

## 3. Materials and Methods

**Figure 1:**
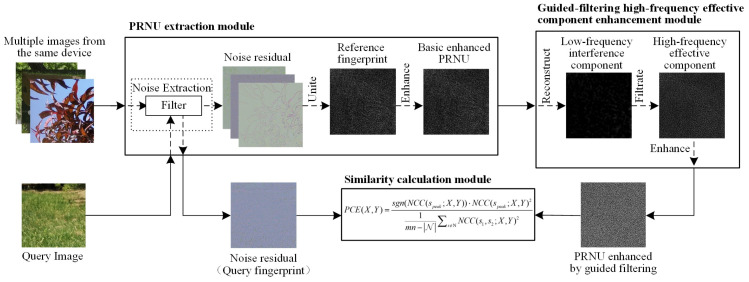
The workflow of PRNU guided-filter enhancement algorithm.

The workflow of the PRNU guided-filter enhancement algorithm proposed is shown in Figure 1. It consists of three main modules: the PRNU extraction module, the guided-filtering high-frequency effective component enhancement module, and the similarity calculation module. Among them, the guided-filtering high-frequency effective component enhancement module is the core module in this paper.

In a real-world open environment SCI task, we begin by using the PRNU extraction module to obtain an initial estimated reference fingerprint. Next, the high-frequency component enhancement module suppresses various low-frequency noise introduced by the post-processing pipeline, further improving PRNU performance. Finally, the similarity calculation module assesses the similarity between the reference and query fingerprints, determining the final image attribution based on a defined threshold. The details of each module are as follows.

### 3.1. PRNU Extraction Module

#### 3.1.1. Noise Extraction Stage

At this stage, noise extraction is performed on the original color or grayscale image to obtain the image noise residual. The noise extracted by the noise extraction algorithm can be explicitly modeled in the following form [2]:

(1) $$W=I_{n}-F(I_{n})=IK+\varepsilon ,$$

where W represents the image noise residual, $I_{n}$ denotes the natural image containing various types of noise, F is the noise extraction algorithm, and I refers to the denoised image, K represents the estimated PRNU, ε encompasses other noise components (mainly additive noise) and random errors. It should be noted that, unless otherwise specified, all matrix operations mentioned in this paper are element-wise operations.

Since the query fingerprint can only be extracted from a single query image, we directly use the noise residual as a substitute. This allows us to approximate PRNU based on the noise information available in that specific image.

#### 3.1.2. Combination Stage

PRNU is quite a weak high-frequency signal that can be easily affected by semantic information and other noise present in the image. Therefore, to extract a relatively pure reference fingerprint, a substantial number of images is needed. Typically, we assume that the additive noise and error terms follow a Gaussian distribution and use the maximum likelihood estimation method to derive the formula for estimating reference fingerprints [41]:

(2) $$\hat{K}=\frac{\sum_{k=1}^{d}I_{k}W_{k}}{\sum_{k=1}^{d}I_{k}^{2}},$$

where d represents the number of noise residuals used to calculate a reference fingerprint. Generally speaking, the larger the number of noise residuals, the higher the quality of the fingerprint. This is because having more samples helps to average out the noise and provides a more accurate estimate of the underlying PRNU.

**Figure 2:**
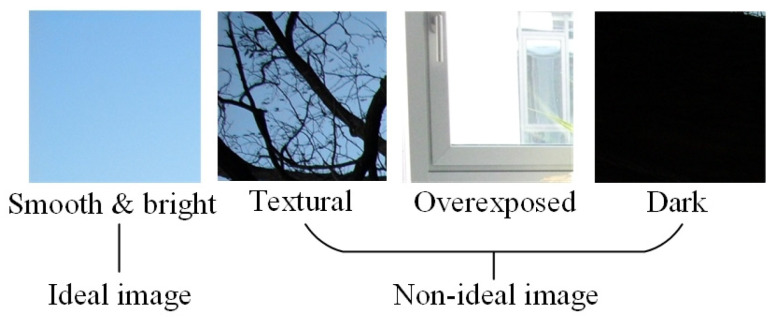
Ideal image and non-ideal image for PRNU estimation.

The extracted noise residuals often contain significant semantic information, which can interfere with PRNU performance. Since PRNU is a type of multiplicative noise intertwined with this semantic content, separating them is challenging. As shown in Figure 2, ideal images for PRNU estimation should be smooth and bright, such as clear blue-sky images. In contrast, images with complex textures or exposure issues are less suitable for PRNU extraction. However, in real-world scenarios, it may be difficult to control the imaging device, making it challenging to obtain enough high-quality images.

#### 3.1.3. Enhancement Stage

PRNU can be affected by low-frequency interference introduced during the post-processing pipeline, including sensor-induced artifacts, demosaicing, compression effects, etc. This is especially noticeable in devices from the same brand, where similar internal image processing leads to comparable fingerprints across different cameras.

Consequently, after the initial extraction of reference fingerprints, further processing is necessary to enhance the effective components and suppress interference. Popular enhancement algorithms include the Removing Shared Components (RSC) method [4] and the Spectrum Equalization Algorithm (SEA) [16]. This paper also analyzes the decorrelation method (DC) proposed in [17].

In the RSC algorithm, a zero-mean operation is applied to each row and column of the reference fingerprint to eliminate periodic artifacts introduced by demosaicing. Wiener filtering in the frequency domain further suppresses non-periodic artifacts. The SEA algorithm smooths the reference fingerprint by removing abnormal peaks in the frequency domain, while the DC method suppresses random artifacts by reducing principal components with eigenvalues exceeding the theoretical variance of the reference fingerprint in the PCA domain.

### 3.2. Guided-Filter High-Frequency Effective Component Enhancement Module

#### 3.2.1. High-Frequency Enhancement Principle Based on Guided Filtering

Guided filtering [42] is a classical edge-preserving smoothing algorithm that reconstructs an image by applying linear transformations to local windows of a guiding image, ensuring that the filtered image closely approximates the target image. This technique has found widespread applications in tasks such as denoising, dehazing, deraining, detail enhancement, and image segmentation. Its fundamental principle is as follows.

For each filter window $w_{k}$, perform the guided filtering operation such that the output image O remains as consistent as possible with the input image I:

(3) $$O_{i}=a_{k}\cdot G_{i}+b_{k},i\in w_{k},$$

where $O_{i}$ and $G_{i}$ represents the pixel values at each position within window $w_{k}$ in the filtered output image and the guided image, respectively. The term $a_{k}$ refers to the filtering parameter corresponding to window $w_{k}$, which determines how closely the filtered image aligns with the guide image, while $b_{k}$ is the bias parameter that adjusts the local intensity offset within the window. These parameters help in preserving the structural details of the image while performing smoothing or enhancement operations.

Based on the minimization of the distance between the filtered image and the target image, the optimization objective is set for each filtering window:

(4) $$\min\sum_{i\in w_{k}}{\left\|a_{k}\cdot G_{i}+b_{k}-I_{i}\right\|}^{2}+\varepsilon {\left\|a_{k}\right\|}^{2},$$

where ε represents the regularization coefficient, which adjusts the coefficient $a_{k}$ to indirectly modify the filtering result; $\left\|\cdot \right\|$ denotes L2 norm.

By taking the partial derivative of the objective function with respect to parameter $a_{k}$ and $b_{k}$ and setting them to zero, the closed-form solution is obtained:

(5) $$a_{k}=\frac{Cov{\left(G,I\right)}^{w_{k}}}{Var{\left(G\right)}^{w_{k}}+\varepsilon },$$

(6) $$b_{k}=mean{\left(I\right)}^{w_{k}}-a_{k}\cdot mean{\left(G\right)}^{w_{k}},$$

where $Cov{\left(\cdot \right)}^{w_{k}},Var{\left(\cdot \right)}^{w_{k}}$, and $mean{\left(\cdot \right)}^{w_{k}}$ represent the covariance, variance, and mean of the specified object within the filter window $w_{k}$, respectively.

Substituting into the original equation, the output image for the filter window $w_{k}$ can be expressed as

(7) $$O_{i}=\sum_{k:i\in w_{k}}\left(mean{\left(I\right)}^{w_{k}}+a_{k}(G_{i}-mean{\left(G\right)}^{w_{k}})\right),$$

where $k:i\in w_{k}$ denotes the ith pixel within the kth filter window $w_{k}$.

Subsequently, the final output image is obtained by applying mean filtering to the computed results from all the windows, which will not be elaborated further. It can be observed that the filtered output image essentially represents a weighted average of the low-frequency components of the original image I and the high-frequency components of the guiding image G, with the weight $a_{k}$ being directly influenced by the regularization parameter ε.

Based on the mechanism of guided filtering, it is evident that both the parameter selection and the final mean filtering operation may lead to image smoothing. Therefore, an additional operation can be performed to enhance the high-frequency components of the image. The formula for this operation is as follows,

(8) $$\hat{O}=\lambda \cdot (I-O)+O,$$

where $\hat{O}$ represents the image after enhancing the high-frequency information and λ indicates the enhancement intensity.

#### 3.2.2. PRNU High-Frequency Effective Component Enhancement

As shown in the experimental analysis in Section 4.3., PRNU contains negligible low-frequency components below 10 Hz, which we identify as low-frequency interference. Meanwhile, components above 10 Hz are recognized as high-frequency effective elements. Based on the guided filtering enhancement principle, we construct a low-frequency fingerprint $K_{low}$ with minimal PRNU content. The difference between the original $K_{raw}$ and low-frequency $K_{low}$ reveals high-frequency components $K_{high}$ that require enhancement $K_{adv}$, controlled by a parameter λ that adjusts the strength of the enhancement.

Using Equation (7), the filtered PRNU is a weighted average of the low-frequency portion of the original fingerprint and the high-frequency portion of the guided fingerprint. By using the fingerprint components below 10 Hz, denoted as $K_{u10}$, as a guide image, guided filtering is applied to $K_{\mathrm{raw}}$ to achieve low-frequency reconstruction, obtaining the desired low-frequency fingerprint $K_{low}$. Since an ideal band-pass filter does not exist in practice, directly using a band-pass filtered result is not recommended; instead, the low-frequency fingerprint should be reconstructed from the original PRNU.

**Figure 3:**
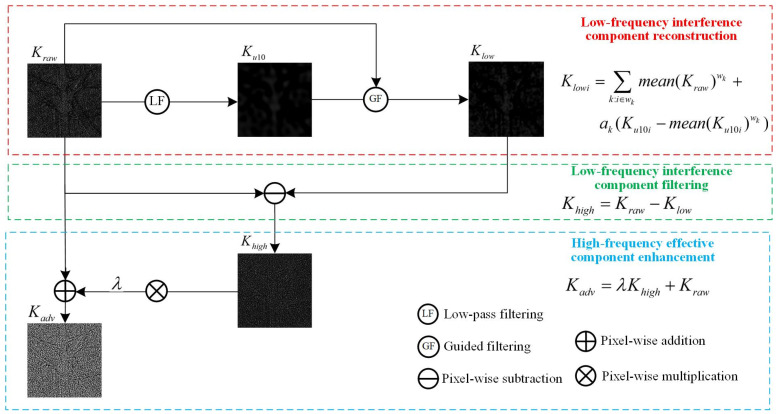
The calculation steps of guided-filtering PRNU high-frequency effective component enhancement.

In summary, we propose a plug-and-play PRNU high-frequency enhancement method, with detailed steps in Equations (9)–(11) and a schematic diagram in Figure 3.

Step 1 Low-frequency interference component reconstruction

Based on guided filtering, PRNU components below the 10 Hz frequency band, denoted as $K_{u10}$, are used as the guide image to reconstruct the original PRNU $K_{raw}$. This results in a low-frequency fingerprint $K_{low}$, which more accurately reflects the true low-frequency inference components of PRNU:

(9) $$K_{lowi}=\sum_{k:i\in w_{k}}mean{\left(K_{raw}\right)}^{w_{k}}+a_{k}(K_{u10i}-mean{\left(K_{u10i}\right)}^{w_{k}}),$$

where $a_{k}=\frac{Cov{\left(K_{u10},K_{raw}\right)}^{w_{k}}}{Var{\left(K_{u10}\right)}^{w_{k}}+\varepsilon }$. It is important to note that $K_{low}$ contains almost no PRNU components and essentially represents low-frequency interference. For clarity in this paper, it is referred to as the “low-frequency fingerprint”.

Step 2 Low-frequency interference component filtering

By filtering out the low-frequency interference component $K_{low}$, the high-frequency effective PRNU component $K_{high}$ can be obtained:

(10) $$K_{high}=K_{raw}-K_{low}.$$

Step 3 High-frequency effective component enhancement

By enhancing the high-frequency effective component $K_{high}$, the final high-frequency enhanced fingerprint $K_{adv}$ can be obtained:

(11) $$K_{high}=\lambda K_{high}+K_{raw}.$$

### 3.3. Similarity Calculation Module

The current mainstream solution in academia for addressing the image provenance problem based on PRNU is the generalized likelihood ratio test [43]. This is framed as a dual-channel hypothesis testing problem. The simplified statistic can be viewed as a form of cosine similarity, commonly referred to as Normalized Cross-Correlation (NCC).

(12) $$H_{0}:K_{1}\neq K_{2},\;H_{1}:K_{1}=K_{2}.$$

(13) $$NCC(s_{1},s_{2};X,Y)=\frac{\sum_{i=1}^{m}\sum_{j=1}^{n}\left(X(i,j)-\bar{X})(Y(i+s_{1},j+s_{2})-\bar{Y}\right)}{\left\|X-\bar{X}\right\|\left\|Y-\bar{Y}\right\|},$$

where X=W, $Y=I\hat{K}$; m and n represents the height and width of the query fingerprints (i.e., the noise residuals), respectively, $s_{1}$ and $s_{2}$ denote the horizontal and vertical step sizes used when comparing query fingerprints with reference fingerprints, respectively. In this paper, both $s_{1}$ and $s_{2}$ are set to a length of 1 pixel.

To further enhance the robustness of this SCI method, the Peak to Correlation Energy (PCE) has been proposed [44]:

(14) $$PCE(X,Y)=\frac{sgn(NCC(s_{peak};X,Y))\cdot NCC{\left(s_{peak};X,Y\right)}^{2}}{\frac{1}{mn-\left|N\right|}\sum_{s\notin Ν}NCC{\left(s_{1},s_{2};X,Y\right)}^{2}},$$

where N represents the narrow neighborhood that achieves the maximum value of NCC (i.e., $NCC(s_{peak};X,Y)$) and sgn(⋅) denotes the sign function. In this paper, N is set to a rectangular area of size 11 × 11.

The calculation steps of PCE indicate that this statistic amplifies the practical significance of the correlation metric NCC. Specifically, for scenarios that conform to the $H_{1}$ hypothesis, where the images from the source device are compared with their corresponding PRNU fingerprints, the computed PCE value will be substantially high. Conversely, in scenarios that conform to the $H_{0}$ hypothesis, the calculated PCE value will approach zero.

## 4. Experiment and Discussion

In this section, we provide a detailed explanation and analysis of the experimental design and results. To measure the actual frequency range of PRNU and to validate the performance of the proposed enhancement module in various scenarios, we designed two main experiments: PRNU frequency band analysis experiments and PRNU enhancement experiments. Additionally, an operation time analysis of the algorithm is presented at the end.

### 4.1. Experimental Environment and Data Preparation

Regarding the experimental hardware configuration, we use an Intel Core i7-9750H CPU (Intel Corporation, Santa Clara, CA, USA) with a base frequency of 2.60 GHz. For the software configuration, we utilize MATLAB R2023a scientific computing tool.

In this paper, we select publicly available Dresden [45] and Daxing [46] digital image forensics datasets for our experiments. The Dresden dataset consists of 16,961 JPEG images and 1491 RAW images captured by 74 cameras across 25 models from 14 brands, making it one of the most widely used datasets for SCI experiments. The Daxing dataset includes 43,400 JPEG images captured by 90 commercial smartphones across 22 models from 5 brands, representing the largest smartphone forensics dataset to date. Given the differences in imaging characteristics between cameras and smartphones, using both datasets lends greater credibility to the experimental results. Additionally, both datasets include images from various real-world scenarios, accounting for different lighting conditions and levels of texture complexity. This helps simulate forensic tasks in realistic environments.

To facilitate the experiments and evaluate the algorithm’s performance across different image resolutions, each image in the two datasets is uniformly cropped from the top-left corner into three sizes: 128 × 128, 256 × 256, and 512 × 512. This effectively creates images with three distinct resolutions, forming the experimental dataset used in this study. As mentioned in Section 3.1, flat-field images like blue sky are ideal for extracting PRNU. However, in real-world scenarios, most images contain complex textures and may include overexposed or underexposed areas. Therefore, to closely simulate real-world conditions, for each imaging device in the two datasets, the first 150 images (in natural order) are selected. The first 50 images are used for reference fingerprint estimation, while the remaining 100 images are utilized for query fingerprint extraction.

### 4.2. Evaluation Metrics

We select the area under the ROC curve (AUC), the true positive rate at a false positive rate of 10^−3^ (TPR@FPR10^−3^), and the Kappa statistic as evaluation metrics to assess the performance of PRNU. These metrics are chosen to provide a comprehensive evaluation of the detection capabilities and consistency of the PRNU enhancement.

For each imaging device in two datasets, the first 50 images (in natural order) are selected to estimate the reference fingerprint for that device. Then, the extracted query fingerprints from each of the remaining 100 images for each device are compared individually with the corresponding reference fingerprints. PCE values are finally calculated for each comparison, resulting in a PCE matrix that reflects the correlation between the reference and query fingerprints for performance evaluation.

#### 4.2.1. AUC and TPR@FPR10^−3^

Using the PCE matrix, we can calculate the True Positive Rate (TPR) and False Positive Rate (FPR) at various thresholds to generate the corresponding ROC curves. From these, we derive the AUC and TPR@FPR10^−3^ values.

(15) $$TPR=\frac{TP}{TP+FN},\;FPR=\frac{FP}{FP+TN},$$

where TP (True Positive), FN (False Negative), FP (False Positive), and TN (True Negative) represent the following: TP is the number of images correctly classified as belonging to the same devices (true matches), FN is the number of images incorrectly classified as belonging to different devices (missed matches), FP is the number of images incorrectly classified as belonging to the same devices (false matches), and TN is the number of images correctly classified as belonging to different devices (true non-matches).

#### 4.2.2. Kappa Statistic

The Kappa statistic is a useful metric for assessing classification consistency. In the PCE matrix, the highest value in each group is designated as the positive sample, while the remaining entries are classified as negative. By comparing these classifications with expected outcomes, we can derive the TOP-1 confusion matrix. From this confusion matrix, the Kappa statistic is then calculated using the formula shown below:

(16) $$\kappa =\frac{p_{o}-p_{e}}{1-p_{e}},$$

where $p_{o}$ is the observed agreement (the proportion of correctly classified images), $p_{e}$ is the expected agreement by chance. We can calculate them, respectively, by

(17) $$p_{o}=\frac{TP+TN}{TP+FP+TN+FN},$$

(18) $$p_{e}=\frac{\left(TP+FN)(TP+FP)+(FP+TN)(FN+TN\right)}{{\left(TP+FP+TN+FN\right)}^{2}}.$$

### 4.3. PRNU Frequency Band Analysis Experiment

As a kind of pattern noise generated during the imaging process, PRNU is regarded as a high-frequency signal [2]. However, research to date has not publicly analyzed its actual frequency range. Therefore, we employ an ideal band-pass filter based on Fourier transform and conduct a band-by-band analysis of PRNU.

#### 4.3.1. Visualization Analysis

**Figure 4:**
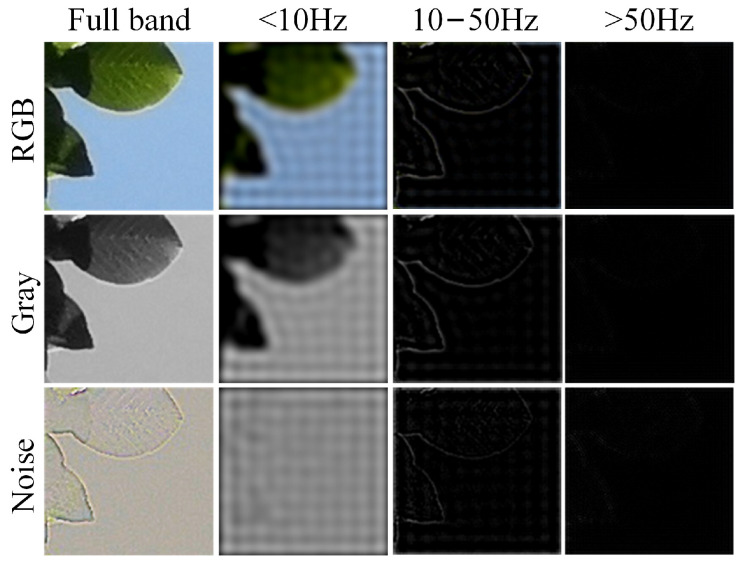
Image segmentation results across different frequency bands. The rows from top to bottom display the color image, grayscale image, and noise image, respectively. From left to right, the columns represent the full-band frequency image, the image filtered to frequencies below 10 Hz, the 10–50 Hz frequency band image, and the image filtered to frequencies above 50 Hz.

PRNU appears as pattern noise within images, but due to its lack of semantic content, direct analysis is challenging. To visually examine its characteristics across different frequency bands, we selected an image containing both complex textured areas (high-frequency regions) and smooth areas (low-frequency regions), clearly separated from each other. We present a frequency-by-frequency visualization of the color image, grayscale image, and noise residuals extracted using noise extraction algorithms. To capture image noise that contains as many PRNU components as possible, we adopt the method from [12] using a Densely connected Hierarchical Denoising Network (DHDN) for noise extraction. The visualization results are displayed in Figure 4.

From the analysis of Figure 4, it is evident that the noise extraction algorithm primarily functions as a high-pass filter, removing the low-frequency semantic information below 10 Hz from the original image. Simultaneously, the 10–50 Hz frequency band in the noise image retains most of the high-frequency details of the image. All three types of images show that the high-frequency band above 50 Hz contains almost no semantic information. Based on the mechanism of PRNU generation, it is known that PRNU, as a high-frequency multiplicative noise, is embedded within the image. Therefore, it can be inferred that its frequency range is likely above 10 Hz.

#### 4.3.2. Experimental Analysis

The PRNU frequency band analysis experiments are conducted on the Dresden and Daxing datasets, with each dataset using an image resolution of 128 × 128 pixels. The experiments comprised four groups: “Baseline”, “RSC”, “SEA”, and “DC”. During the noise residual extraction stage, the DHDN noise extraction algorithm is employed for the three experimental groups. In the enhancement stage, “Baseline” indicates no enhancement algorithm is applied, while “RSC”, “SEA”, and “DC” represent three of the most commonly used enhancement algorithms. The segmented frequency bands of the PRNU (i.e., reference fingerprint) are directly utilized in SCI experiments, and the presence of the PRNU components in each frequency band is indirectly inferred through the observation of performance metrics.

**Figure 5:**
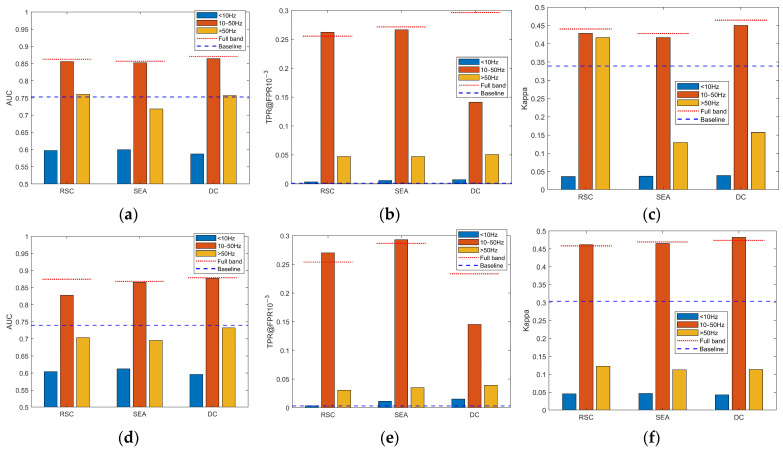
Results of the PRNU frequency band analysis experiments. The first row (**a**–**c**) shows the results on the Dresden dataset, while the second row (**d**–**f**) presents those on the Daxing dataset. In each subfigure, “Full band” represents the use of full-band PRNU in the SCI experiments.

In the actual experiments, a step size of 5 Hz is employed for the frequently-by-frequently analysis. It is observed that the values of the corresponding metrics in the 0–5 Hz and 5–10 Hz frequency bands are extremely low, while each step interval within the 10–50 Hz band exhibited significantly higher corresponding metric values. In contrast, the overall metric values in the frequency band above 50 Hz are relatively low. To conserve space and highlight the key findings, the experimental results have been summarized and consolidated, as presented in Figure 5.

The experimental results presented in Figure 5 indicate that for both Dresden and Daxing datasets, as well as for the RSC, SEA, and DC enhancement schemes, the reference fingerprints within the 10–50 Hz frequency band achieve performance levels comparable to those of the full-frequency band ones. Furthermore, it is noteworthy that the metrics corresponding to the low-frequency band below 10 Hz are extremely low, while the metrics for the high-frequency band above 50 Hz are significantly lower than those in the 10–50 Hz band yet still higher than those in the sub-10 Hz band.

Combining the results of the experimental analysis, it is evident that the PRNU, as a high-frequency multiplicative pattern noise, primarily exists in the frequency band above 10 Hz in images. Therefore, enhancing this specific frequency band can lead to the design of a plug-and-play PRNU enhancement algorithm aimed at achieving further improvements in its performance.

### 4.4. PRNU Enhancement Experiment

Following the algorithm hyperparameter analysis in Section 4.4.4, the guided filtering window diameter r, regularization coefficient ε, and enhancement coefficient λ are set to 5, 0.01, and 5, respectively. To comprehensively evaluate the proposed algorithm’s effectiveness and robustness, we conduct experiments on non-JPEG and JPEG compression scenes, as well as an image texture complexity analysis experiment.

#### 4.4.1. Non-JPEG Compression Scene Enhancement Experiments

To thoroughly evaluate the performance of the proposed algorithm, experiments are conducted in non-JPEG compression scenarios using Dresden and Daxing datasets. The analysis encompassed three image resolutions: 128 × 128, 256 × 256, and 512 × 512, along with three basic PRNU enhancement algorithms: RSC, SEA, and DC. Additionally, various experimental scenarios are established by combining these basic schemes with the approaches from [15] and the proposed methods, respectively.

Specifically, for each image resolution in both datasets, ten experimental setups are created: “Baseline”, “RSC”, “SEA”, “DC”, “RSC + HF”, “SEA + HF”, “DC + HF”, “RSC + Ours”, “SEA + Ours”, and “DC + Ours”. During the noise residual extraction stage, all ten experiments utilized the DHDN noise extraction algorithm. For the enhancement stage, “Baseline”, “RSC”, “SEA”, and “DC” retained the same meanings as previously defined. The experiments “RSC + HF”, “SEA + HF”, and “DC + HF” apply the algorithm from [15] on top of the RSC, SEA, and DC base enhancement schemes, respectively. Meanwhile, “RSC + Ours”, “SEA + Ours”, and “DC + Ours” incorporate the proposed algorithm as a plug-and-play module into the RSC, SEA, and DC enhancement schemes. The experimental results on the Dresden dataset are summarized in Table 1, while those on the Daxing dataset are placed in Table A1, Appendix A, for better readability.

Since the algorithm proposed in [15] does not clearly define the specific range of PRNU low-frequency components to be removed, its hyperparameters cannot be generalized to all scenarios. The results in Table 1 and Table A1 demonstrate that this approach still leads to the degradation of the high-frequency effective components of PRNU, thus negatively affecting its performance. In contrast, the enhancement algorithm proposed in this paper explicitly identifies the range of low-frequency interference components that need to be removed, resulting in improved PRNU performance across all cases.

Given that PRNU lacks semantic information, we further utilize spectrum visualization to demonstrate the effectiveness of the proposed algorithm. Using the Dresden experimental dataset, we take the imaging device named “Agfa_DC-504_0” (the first device selected in order) and its 128 × 128 resolution images as an example. The frequency spectrum of the PRNU is processed by all 10 different enhancement schemes. For better observation and analysis, the most effective frequency range of PRNU between 10–50 Hz is highlighted in red.

**Figure 6:**
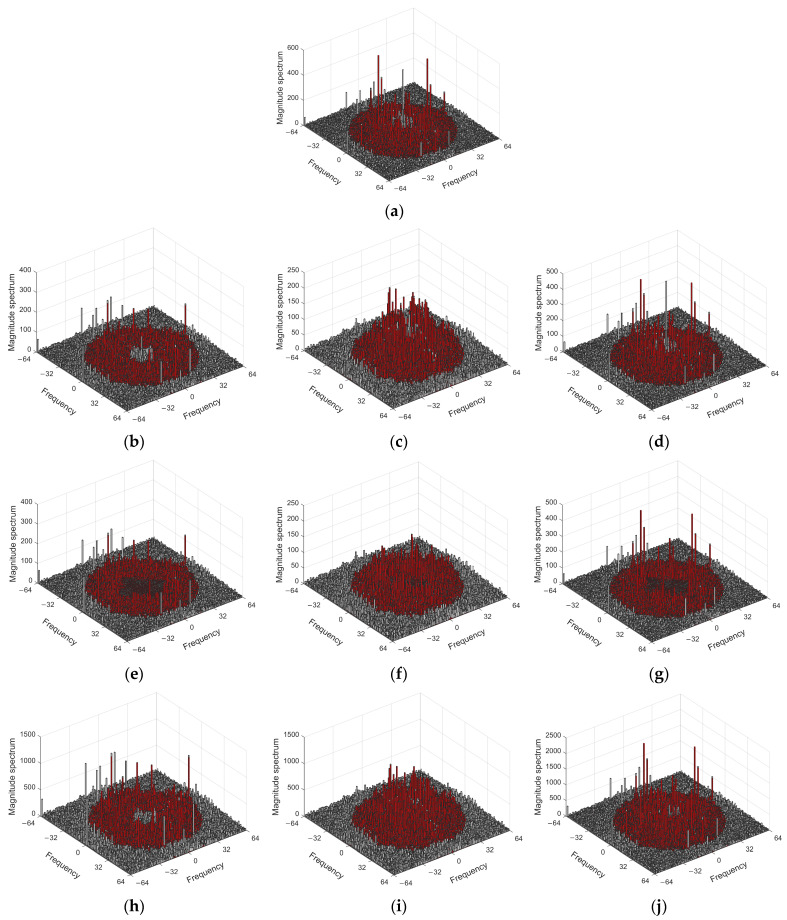
PRNU spectrum under different enhancement schemes. (**a**) “Baseline” (non-enhancement); (**b**) “RSC” enhancement scheme; (**c**) “SEA” enhancement scheme; (**d**) “DC” enhancement scheme; (**e**) “RSC + HF” enhancement scheme; (**f**) “SEA + HF” enhancement scheme; (**g**) “DC + HF” enhancement scheme; (**h**) “RSC + Ours” enhancement scheme; (**i**) “SEA + Ours” enhancement scheme; (**j**) “DC + Ours” enhancement scheme.

As shown in Figure 6a, the frequency spectrum of PRNU without enhancement displays multiple local peaks, distributed in both the low-frequency range (below 10 Hz) and the high-frequency range (above 10 Hz), with interference-induced peaks significantly affecting PRNU performance. Figure 6b demonstrates that the RSC enhancement scheme provides only limited suppression of low-frequency interference below 10 Hz.

Figure 6c,d shows that, while the SEA and DC enhancement schemes strengthen high-frequency PRNU components above 10 Hz, they do not effectively suppress low-frequency interference below this threshold. Therefore, each of these methods has room for improvement.

While the enhancement algorithm proposed in [15] enhancement algorithm recognizes this issue, it does not clearly define the frequency range of the low-frequency interference components. Instead, it treats the problem as a black-box algorithm requiring parameter tuning, which makes it unsuitable for all cases. As seen in Figure 6e–g, this approach removes the low-frequency interference but also damages the effective high-frequency components of the PRNU, resulting in a decrease in performance.

In contrast, our algorithm, based on extensive experiments, explicitly identifies the frequency ranges for both the low-frequency interference and the high-frequency effective components of the PRNU. As shown in Figure 6h–j, our algorithm not only suppresses the low-frequency interference but also significantly enhances the high-frequency components, further improving the performance of PRNU.

In summary, the proposed algorithm demonstrates effectiveness across images with different resolutions and various base enhancement algorithms, validating the utility of the module. However, the experimental results also indicate that not all performance metrics show improvement in every scenario.

Upon analysis, this can be attributed to two main factors: first, the inherent variability and fluctuation in the metrics themselves; second, the use of a single set of parameters across all scenarios, which may not be the optimal set for every case. Additionally, since most of the algorithm’s parameters are continuous variables, the step size used for parameter optimization may have been too large, potentially leading to missed optimal solutions.

#### 4.4.2. JPEG Compression Scene Enhancement Experiments

Reference [47] points out that social platforms apply minimal compression to standard and small digital images, but for larger images, the compression rate exceeds 80%, significantly reducing PRNU performance. In this paper, images with a resolution of 128 × 128 are used to evaluate four JPEG compression scenarios with quality factors of 90, 80, 70, and 60. Ten sets of experiments are conducted for each scenario, with nine sets of experiments designed in the same manner as previously described. The experimental results on the Dresden dataset are summarized in Table 2, while those on the Daxing dataset are placed in Table A2, Appendix A, for better readability.

Analyzing the experimental results shown in Table 2 and Table A2, we observe that under different JPEG compression scenarios, the scheme proposed in [15] impaired the high-frequency effective components of PRNU, thus failing to enhance the PRNU. In contrast, the algorithm proposed in this paper consistently achieves performance enhancement of PRNU across all scenarios, thereby validating its robustness.

Additionally, we observe that in a few cases, the proposed algorithm does not improve all performance metrics of PRNU as well. Considering the impact of JPEG compression on the high-frequency components of PRNU, this could be another contributing factor, alongside the reasons discussed in Section 4.4.1.

#### 4.4.3. The Effect of Image Texture Complexity Analysis Experiment

To confirm the effectiveness of our proposed algorithm in general scenarios, we conduct reference fingerprint enhancement experiments on natural images in Section 4.4.1. To further evaluate the algorithm’s sensitivity to image texture complexity, we conduct additional tests on both flat field and textured images in this subsection.

First, we reorganized the Daxing dataset with 128 × 128 resolution images. Specifically, we use Entropy as a measure of image texture complexity (higher Entropy indicates more complex textures, and vice versa) to rank the 150 images from each imaging device in the dataset. From each device’s set, we selected the 50 images with the most complex textures and the 50 smoothest images to form two sub-datasets, T for complex textures and F for flat fields, to be used for reference fingerprint extraction. The remaining 50 images formed dataset S, designated for SCI experiments.

For comparison, we use a non-enhanced PRNU extraction as the “Baseline” and the RSC enhancement method as a basic enhancement technique. The experimental results are shown in Table 3.

As shown in Table 3, the performance of reference fingerprints extracted from flat-field images is significantly better than that of fingerprints extracted from textured images, indicating that complex textures notably impact PRNU. However, our proposed algorithm improves PRNU performance in both scenarios. Specifically, for fingerprints extracted from textured images, the TPR@FPR10^−3^ increased from 0.3689 to 0.5551, significantly closing the performance gap with ideal fingerprints extracted from flat-field images, which achieved a metric value of 0.5613. These results demonstrate the robustness of the proposed algorithm against varying image textures.

#### 4.4.4. Algorithm Hyper-Parameter Analysis Experiment

The proposed algorithm involves several key hyper-parameters, including the guided filter window diameter r, the regularization coefficient ε, and the enhancement factor λ. Upon analysis in Section 3.2.1, it can be observed that as r and ε increase, the high-frequency information contained in the resulting low-frequency fingerprint $K_{low}$ decreases. However, this comes at the cost of reduced accuracy in the reconstruction of $K_{low}$. Since $K_{low}$ deviates from the actual low-frequency components of the original fingerprint $K_{raw}$, the corresponding high-frequency fingerprint $K_{high}$ also deviates from the true high-frequency components of PRNU, thereby affecting the final enhancement effect. As for λ, a larger value leads to a higher degree of high-frequency enhancement for PRNU, but it also results in increased computational complexity. Beyond a certain point, the gain from enhancement diminishes and may even introduce interference factors, which can negatively impact the overall performance. Therefore, the specific hyper-parameter settings must be determined based on a comprehensive analysis of extensive experimental results.

**Figure 7:**
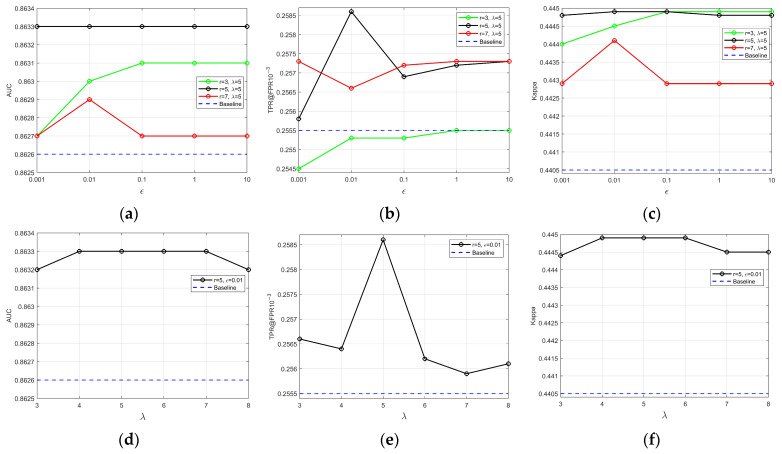
The results of the algorithm hyper-parameter analysis experiment. (**a**–**c**) present the experimental analysis results for varying r and ε values with λ fixed at 5, while (**d**–**f**) show the results for varying λ values with r and ε fixed at 5 and 0.01, respectively.

We conducted hyper-parameter analysis experiments on 128 × 128 resolution images from the Dresden dataset, using the RSC enhancement scheme as the “Baseline”. Based on empirical insights from [42], we set λ to 5 and explored various values for r and ε. As shown in Figure 7a–c, when r and ε are set to 5 and 0.01, respectively, the best results are achieved in terms of AUC, TPR, and Kappa, with values of 0.8633, 0.2586, and 0.4449, respectively. Next, we fixed r and ε at 5 and 0.01 and experimented with different values of λ. As illustrated in Figure 7d–f, the optimal PRNU performance is achieved when λ is set to 5. Notably, when r and ε are set appropriately, PRNU performance improves consistently, regardless of the specific value of λ. This further supports the approach of reconstructing and selectively filtering PRNU’s confirmed low-frequency components rather than relying solely on hyperparameter tuning to filter them out directly [15]. This approach enhances the proposed algorithm’s ability to generalize effectively in out-of-distribution scenarios [48].

Ultimately, the optimal hyper-parameter combination is determined to be {r=5,ε=0.01,λ=5}.

### 4.5. Running Time Analysis

Due to the variability in algorithm execution time, we repeat each experiment 10 times and take the arithmetic mean to statistically analyze the average running time per reference fingerprint under different enhancement schemes. Analyzing the results in Table 4, it can be observed that the proposed enhancement algorithm is independent of the choice of image dataset and the underlying enhancement algorithm and is only related to image resolution. Since the proposed algorithm not only filters out the low-frequency interference components of PRNU but also enhances the high-frequency effective components, the execution time is slightly higher than that of the method presented in [15]. Considering various scenarios, the execution time of the proposed algorithm falls between the RSC and SEA enhancement algorithms and is significantly lower than that of the DC enhancement algorithm, thereby validating the efficiency of the proposed method.

## 5. Conclusions

In this paper, we conducted experimental analyses that reveal PRNU primarily resides in frequency bands above 10 Hz. Low-frequency components below 10 Hz are identified as interference, while components above this threshold represent effective high-frequency PRNU signals. Based on these insights, we designed a guided filtering PRNU enhancement algorithm as a plug-and-play module, which can be integrated with existing enhancement techniques. With low computational complexity, this algorithm enhances PRNU performance, even when affected by JPEG compression and complex image textures.

Beyond SCI tasks, our method holds promise for image integrity authentication and identity verification applications. It can be seamlessly integrated with future algorithms, demonstrating significant potential for use in multimedia forensics. Additionally, this algorithm shows a particular promise for improving image authenticity verification in cybersecurity systems, where accurate image authentication is critical.

However, given the diverse demands of real-world applications, the chosen hyper-parameters may not be universally optimal. A promising path for future work is the development of an adaptive PRNU high-frequency component enhancement approach tailored to different scenarios. This adaptive approach would ensure robust PRNU performance across various application contexts, further extending the utility of the proposed method in real-world cybersecurity and digital forensics.

## Figures and Tables

**Table 1:** Non-JPEG compression scene enhancement experiments on the Dresden dataset.

Resolution	Enhancement Scheme	AUC	TPR@FPR10^−3^	Kappa
128 × 128	Baseline	0.7530	0.0011	0.3389
	RSC	0.8626	0.2555	0.4405
	RSC + HF	0.8324	0.1958	0.3617
	RSC + Ours	**0.8633**	**0.2586**	**0.4449**
	SEA	**0.8570**	0.2715	0.4280
	SEA + HF	0.8216	0.2219	0.3437
	SEA + Ours	**0.8570**	**0.2727**	**0.4319**
	DC	0.8710	**0.2964**	0.4645
	DC + HF	0.8420	0.2153	0.3786
	DC + Ours	**0.8728**	0.2928	**0.4684**
256 × 256	Baseline	0.7423	0.0011	0.4337
	RSC	0.9234	0.4520	0.6671
	RSC + HF	0.9024	0.3800	0.5912
	RSC + Ours	**0.9250**	**0.4546**	**0.6704**
	SEA	0.9233	**0.5034**	0.6556
	SEA + HF	0.8984	0.4459	0.5725
	SEA + Ours	**0.9234**	0.5011	**0.6611**
	DC	0.9279	0.4849	0.6823
	DC + HF	0.9071	0.3942	0.5973
	DC + Ours	**0.9307**	**0.4911**	**0.6868**
512 × 512	Baseline	0.7083	0.0014	0.4760
	RSC	0.9631	0.6518	0.8299
	RSC + HF	0.9489	0.5941	0.7658
	RSC + Ours	**0.9642**	**0.6576**	**0.8308**
	SEA	0.9629	0.7115	0.8205
	SEA + HF	0.9475	0.6692	0.7651
	SEA + Ours	**0.9640**	**0.7215**	**0.8273**
	DC	0.9575	0.6362	0.8171
	DC + HF	0.9479	0.5672	0.7559
	DC + Ours	**0.9640**	**0.6559**	**0.8356**

**Table 2:** JPEG compression scene enhancement experiments on Dresden dataset.

Quality Factor	Enhancement Scheme	AUC	TPR@FPR10^−3^	Kappa
90	Baseline	0.7464	0.0011	0.3322
	RSC	0.8552	0.2499	0.4262
	RSC + HF	0.8227	0.1858	0.3425
	RSC + Ours	**0.8560**	**0.2522**	**0.4299**
	SEA	0.8529	**0.2664**	0.4221
	SEA + HF	0.8158	0.2127	0.3321
	SEA + Ours	**0.8531**	0.2659	**0.4232**
	DC	0.8648	0.2858	0.4497
	DC + HF	0.8334	0.2084	0.3664
	DC + Ours	**0.8666**	**0.2878**	**0.4577**
80	Baseline	0.7306	0.0011	0.3132
	RSC	0.8444	0.2295	0.4001
	RSC + HF	0.8098	0.1704	0.3131
	RSC + Ours	**0.8453**	**0.2318**	**0.4026**
	SEA	0.8438	0.2424	0.3956
	SEA + HF	0.8043	0.1886	0.3056
	SEA + Ours	**0.8439**	**0.2431**	**0.3988**
	DC	0.8553	0.2724	0.4288
	DC + HF	0.8213	0.1897	0.3359
	DC + Ours	**0.8573**	**0.2726**	**0.4326**
70	Baseline	0.7365	0.0011	0.3073
	RSC	0.8438	0.2274	0.3892
	RSC + HF	0.8088	0.1603	0.2995
	RSC + Ours	**0.8450**	**0.2296**	**0.3956**
	SEA	**0.8428**	**0.2404**	0.3874
	SEA + HF	0.8017	0.1774	0.2908
	SEA + Ours	**0.8428**	0.2373	**0.3897**
	DC	0.8556	0.2696	0.4230
	DC + HF	0.8209	0.1846	0.3256
	DC + Ours	**0.8574**	**0.2714**	**0.4277**
60	Baseline	0.7376	0.0009	0.3006
	RSC	0.8355	0.2091	0.3669
	RSC + HF	0.7994	0.1497	0.2818
	RSC + Ours	**0.8365**	**0.2112**	**0.3713**
	SEA	0.8354	**0.2278**	0.3774
	SEA + HF	0.7944	0.1623	0.2807
	SEA + Ours	**0.8356**	**0.2278**	**0.3804**
	DC	0.8488	**0.2566**	0.4057
	DC + HF	0.8135	0.1707	0.3113
	DC + Ours	**0.8505**	**0.2564**	**0.4113**

**Table 3:** The results of the effect of image texture complexity analysis experiment.

Enhancement Scheme	AUC		TPR@FPR10^−3^		Kappa	
	F	T	F	T	F	T
Baseline	0.8477	0.7996	0.2211	0.0044	0.5681	0.4229
RSC	0.9162	0.8784	0.5544	0.3689	0.6649	0.5899
RSC + Ours	**0.9248**	**0.8787**	**0.5613**	**0.5551**	**0.6676**	**0.5942**

**Table 4:** Running time comparison (unit: ms).

Dataset	Enhancement Scheme	Resolution		
		128 × 128	256 × 256	512 × 512
Dresden	RSC	1.25	3.48	19.56
	RSC + HF	3.58	10.19	46.89
	RSC + Ours	4.43	18.06	108.49
	SEA	9.57	14.83	72.61
	SEA + HF	10.40	20.03	97.03
	SEA + Ours	11.70	28.92	132.01
	DC	47.49	191.23	765.68
	DC + HF	49.11	198.77	790.65
	DC + Ours	50.53	207.36	844.11
Daxing	RSC	1.26	3.41	19.75
	RSC + HF	3.42	9.85	44.37
	RSC + Ours	4.42	17.55	108.68
	SEA	8.36	14.79	72.65
	SEA + HF	10.58	19.92	95.57
	SEA + Ours	11.23	29.14	131.78
	DC	47.32	192.04	765.63
	DC + HF	49.57	199.49	788.22
	DC + Ours	50.68	210.02	844.19

## Data Availability

The experimental datasets used in this article are all available for free and open access.

## Appendix A

**Table A1:** Non-JPEG compression scene enhancement experiments on Daxing dataset.

Resolution	Enhancement Scheme	AUC	TPR@FPR10^−3^	Kappa
128 × 128	Baseline	0.7395	0.0031	0.3036
	RSC	0.8743	0.2540	0.4583
	RSC + HF	0.8582	0.2096	0.4028
	RSC + Ours	**0.8763**	**0.2558**	**0.4662**
	SEA	0.8682	0.2667	0.4691
	SEA + HF	0.8472	0.2513	0.4101
	SEA + Ours	**0.8683**	**0.2741**	**0.4702**
	DC	0.8788	0.2336	0.4735
	DC + HF	0.8680	0.2042	0.4139
	DC + Ours	**0.8816**	**0.2394**	**0.4738**
256 × 256	Baseline	0.7208	0.0039	0.3891
	RSC	0.9249	0.2704	0.6557
	RSC + HF	0.9170	0.2167	0.5983
	RSC + Ours	**0.9275**	**0.2714**	**0.6639**
	SEA	0.9268	0.3547	0.6748
	SEA + HF	0.9142	0.3020	0.6217
	SEA + Ours	**0.9274**	**0.3514**	**0.6799**
	DC	0.9296	0.2749	0.6666
	DC + HF	0.9266	0.2242	0.6197
	DC + Ours	**0.9327**	**0.2778**	**0.6739**
512 × 512	Baseline	0.6960	0.0046	0.4237
	RSC	0.9563	**0.3372**	0.8081
	RSC + HF	0.9512	0.2614	0.7691
	RSC + Ours	**0.9576**	0.3361	**0.8137**
	SEA	0.9587	**0.4118**	0.8245
	SEA + HF	0.9538	0.3304	0.7947
	SEA + Ours	**0.9588**	0.4112	**0.8278**
	DC	0.9570	0.3242	0.7879
	DC + HF	0.9586	0.2439	0.7655
	DC + Ours	**0.9611**	**0.3279**	**0.8072**

**Table A2:** JPEG compression scene enhancement experiments on the Daxing dataset.

Quality Factor	Enhancement Scheme	AUC	TPR@FPR10^−3^	Kappa
90	Baseline	0.7435	0.0036	0.2863
	RSC	0.8672	0.2398	0.4393
	RSC + HF	0.8476	0.1996	0.3819
	RSC + Ours	**0.8686**	**0.2500**	**0.4462**
	SEA	**0.8632**	0.2590	0.4502
	SEA + HF	0.8402	0.2272	0.3864
	SEA + Ours	0.8629	**0.2591**	**0.4524**
	DC	0.8739	0.2078	0.4531
	DC + HF	0.8610	0.1797	0.3924
	DC + Ours	**0.8762**	**0.2124**	**0.4536**
80	Baseline	0.7079	0.0028	0.2761
	RSC	0.8444	0.2414	0.3880
	RSC + HF	0.8192	0.1957	0.3147
	RSC + Ours	**0.8459**	**0.2457**	**0.3948**
	SEA	**0.8449**	0.2622	0.4088
	SEA + HF	0.8155	0.2314	0.3310
	SEA + Ours	0.8446	**0.2691**	**0.4099**
	DC	0.8540	0.2616	**0.4216**
	DC + HF	0.8357	0.2081	0.3383
	DC + Ours	**0.8564**	**0.2632**	0.4215
70	Baseline	0.6746	0.0022	0.2449
	RSC	0.8246	0.1939	0.3384
	RSC + HF	0.7930	0.1431	0.2582
	RSC + Ours	**0.8272**	**0.1967**	**0.3409**
	SEA	0.8318	**0.2257**	0.3694
	SEA + HF	0.7980	0.1869	0.2800
	SEA + Ours	**0.8321**	0.2256	**0.3704**
	DC	0.8369	**0.2327**	0.3740
	DC + HF	0.8112	0.1724	0.2835
	DC + Ours	**0.8399**	0.2314	**0.3758**
60	Baseline	0.6461	0.0011	0.2148
	RSC	0.8022	0.1524	0.2819
	RSC + HF	0.7628	0.1002	0.2078
	RSC + Ours	**0.8041**	**0.1532**	**0.2862**
	SEA	0.8133	**0.1654**	0.3161
	SEA + HF	0.7705	0.1409	0.2291
	SEA + Ours	**0.8136**	0.1650	**0.3191**
	DC	0.8178	**0.1982**	**0.3204**
	DC + HF	0.7796	0.1290	0.2278
	DC + Ours	**0.8200**	**0.1982**	0.3198
